# The Role of Sirtuin-1 in the Vasculature: Focus on Aortic Aneurysm

**DOI:** 10.3389/fphys.2020.01047

**Published:** 2020-08-25

**Authors:** Enkhjargal Budbazar, Francisca Rodriguez, José M. Sanchez, Francesca Seta

**Affiliations:** ^1^Vascular Biology Section, Boston University School of Medicine, Boston, MA, United States; ^2^Department of Physiology, University of Murcia and Biomedical Research Institute in Murcia (IMIB), Murcia, Spain

**Keywords:** sirtuin-1, aortic aneurysm, oxidative stress, heme oxgenase, vascular disease

## Abstract

Sirtuin-1 (SirT1) is a nicotinamide adenine dinucleotide-dependent deacetylase and the best characterized member of the sirtuins family in mammalians. Sirtuin-1 shuttles between the cytoplasm and the nucleus, where it deacetylates histones and non-histone proteins involved in a plethora of cellular processes, including survival, growth, metabolism, senescence, and stress resistance. In this brief review, we summarize the current knowledge on the anti-oxidant, anti-inflammatory, anti-apoptotic, and anti-senescence effects of SirT1 with an emphasis on vascular diseases. Specifically, we describe recent research advances on SirT1-mediated molecular mechanisms in aortic aneurysm (AA), and how these processes relate to oxidant stress and the heme-oxygenase (HO) system. HO-1 and HO-2 catalyze the rate-limiting step of cellular heme degradation and, similar to SirT1, HO-1 exerts beneficial effects in the vasculature through the activation of anti-oxidant, anti-inflammatory, anti-apoptotic, and anti-proliferative signaling pathways. SirT1 and HO-1 are part of an integrated system for cellular stress tolerance, and may positively interact to regulate vascular function. We further discuss sex differences in HO-1 and SirT1 activity or expression, and the potential interactions between the two proteins, in relation to the progression and severity of AA, as well as the ongoing efforts for translational applications of SirT1 activation and HO-1 induction in the treatment of cardiovascular diseases including AA.

## Role of SirT1 in the Vasculature

Sirtuin-1 (SirT1, mammalian homolog of silent information regulator (Sir2) in yeast) is a nicotinamide adenine dinucleotide (NAD^+^)-dependent class III histone deacetylase (Frye, 2000) and the best characterized member of the sirtuins family in mammalians (Haigis and Sinclair, 2010). Compelling evidence have demonstrated beneficial effects of SirT1 in the cardiovascular system, generally attributed to anti-oxidant (Kawai et al., 2011; Zhou et al., 2011), anti-senescence (Ota et al., 2010a; Thompson et al., 2014; Chen et al., 2016), anti-apoptotic (Hibender et al., 2016; Hou et al., 2019), and anti-inflammatory effects (Sosnowska et al., 2017; D’Onofrio et al., 2018). In the vasculature, SirT1 is expressed in the endothelium, vascular smooth muscle (VSM) and adventitia (Potente et al., 2007; Miyazaki et al., 2008; Li et al., 2011; Fry et al., 2015; Chen et al., 2016; Ling et al., 2017). SirT1 is localized mostly in the nucleus where it regulates gene transcription, in a cell-specific manner, by deacetylating histone 3 (H3) and transcription factors, such as forkhead box O (FOXOs), nuclear factor kB (NF-kB), tumor protein 53 (p53), peroxisome proliferator-activated receptor-γ co-activator-1α (PPARγ), and the DNA repair protein Ku70 (Brunet et al., 2004; Michishita et al., 2005; Jeong et al., 2007; Chung et al., 2010; Zeng et al., 2013; Sosnowska et al., 2017). Specifically, activation of NF-kB and p53 promotes inflammation, apoptosis, senescence, and oxidant stress in animal models of aortic aneurysm (AA; Gomez et al., 2013; Leeper et al., 2013; Chen et al., 2016; Moran et al., 2017) while activation of FOXOs and PPARγ reverses these processes by opposing pro-inflammatory and apoptotic factors in animal and human studies (Jones et al., 2009; Oellerich and Potente, 2012; Radak et al., 2013; Motoki et al., 2015; Tai et al., 2016; Lu et al., 2020). In addition to gene transcription, SirT1 regulates the activity of several proteins, such as endothelial nitric oxide synthase (eNOS), which is activated to produce the vasoprotective mediator nitric oxide (NO), upon SirT1-mediated deacetylation of lysines 496 and 506 in the eNOS calmodulin-binding domain (Mattagajasingh et al., 2007). Conversely, NO is able to stimulate SirT1 expression and activity via a positive feedback mechanism, at least in the settings of statin- and cilostazol-induced SirT1 expression, which is prevented by the eNOS inhibitor L-NAME (Ota et al., 2008, 2010a,b; Man et al., 2019).

SirT1 is essential for VSM structural and functional homeostasis (Li et al., 2011; Gorenne et al., 2013; Fry et al., 2015; Chen et al., 2016). Physiological cyclic stretch promotes VSM contractile properties via SirT1/FOXO3a, thus maintaining vasoconstriction (Huang et al., 2015). Interestingly, lack of SirT1 in VSM-specific SirT1-ablated mice does not induce any vascular functional impairment *per se* (Fry et al., 2015). However, in response to pro-inflammatory and pro-oxidant stressors, such as angiotensin II (angII) infusion, arterial ligation, hyperlipidemic apoE^–/–^ genetic background, aging and a diet rich in fat and sucrose, VSM SirT1 is absolutely essential to prevent maladaptive arterial remodeling that leads to vascular disease, such as atherosclerosis (Gorenne et al., 2013), aortic dissection (Fry et al., 2015), and arterial stiffness (Fry et al., 2016). Differential effects of SirT1 expression or activity levels in the vasculature are uniquely dependent on disease type, stage and interacting factors and thoroughly reviewed elsewhere (D’Onofrio et al., 2018; Man et al., 2019). In this review, we will focus on yet another role of SirT1 in vascular disease, namely aortic aneurysm, which only recently started to be appreciated.

## SirT1 in Aortic Aneurysm

Aortic aneurysms are abnormal aortic enlargements which can develop in the thoracic (TAA) or abdominal (AAA) regions. Although the pathogenesis behind different forms of TAA and AAA may differ greatly, a combination of genetic predisposition and hypertension, particularly in Marfan’s and related syndromes, generally contribute to the development of TAA prone to dissections, while smoking, male gender and diabetes are the major risk factors for AAA prone to ruptures. Overall, the most troublesome aspect of AA (TAA and AAA), particularly in non-syndromic and idiopathic forms, is that they often remain clinically undiagnosed until the aortic wall dissects or ruptures, potentially causing sudden death (Davies, 1998; Mody et al., 2014). Currently, treatment options against these potentially lethal vascular conditions are limited to blood pressure control and surgical repair, for which the risk of mortality remains high at 30–50%, depending on the repair method employed (Virani et al., 2020); therefore, novel therapeutic targets are urgently needed. We have recently shown that mice with VSM-specific deletion of SirT1 have a drastically increased mortality (70%) in response to angII infusion due to aortic wall dissection, particularly in the thoracic region, which resulted from excess oxidant production and oxidant-stimulated matrix metalloproteinases (MMPs) activation (Fry et al., 2015). Consistent with these findings, VSM-specific overexpression of SirT1 protects aged mice on apoE-deficient genetic background, against angII-induced AAA and rupture by opposing vascular senescence and inflammation (Chen et al., 2016). Moreover, calorie restriction, which is known to activate SirT1 (Guarente, 2013), prevents angII-induced AAA through SirT1-dependent deacetylation of H3 at lysine 9 on the MMP2 gene promoter, which downregulates MMP2 and subsequent elastin fragmentation in the aortic wall (Liu et al., 2016).

Furthermore, endothelial SirT1 has been shown to counteract the deleterious effects of angII on AA formation, believed to be driven mainly by endothelial, but not VSM, angII type 1α receptors (ATR1α) (Rateri et al., 2011). In a model of Marfan’s syndrome (fibrillin-1 mutant mice, Fbn1^mgR/mgR^), deletion of endothelial ATR1α decreased the incidence of TAA (Galatioto et al., 2018). Since angII administration is known to downregulate SirT1 expression and activity in aortic endothelial cells (Marampon et al., 2013), these findings suggest that angII could accelerate the development of TAA in renin-dependent hypertensive Marfan individuals by suppressing SirT1/eNOS and impairing endothelial function.

It is worth mentioning that SirT1 in hematopoietic cells has recently emerged as an important mediator of AA formation. Macrophage-specific deficiency of SirT1 increased the incidence and exacerbated disease severity in a mouse model of angII-induced AAA, by increasing the pro-inflammatory inducible nitric oxide synthase, a marker of M1 macrophages, while decreasing arginase and mannose receptor, two markers of M2 macrophages. On the contrary, SirT1 overexpression in macrophages, achieved by adenoviral transfection, had an opposite effect (Zhang et al., 2018). Overall, multiple studies indicate that SirT1 in variety of vascular cells is essential for the maintenance of vascular wall integrity and to prevent AA.

## SirT1 and Oxidative Stress in Aortic Aneurysm

An imbalance between the production of reactive oxygen species (ROS) and the cellular anti-oxidant systems, defined as oxidant stress, is a hallmark of vascular diseases, including atherosclerosis and diabetic endothelial dysfunction (Brown and Griendling, 2015; Quintana and Taylor, 2019). A role of oxidant stress in the development and progression of AA has been described recently (Quintana and Taylor, 2019). In human and animal studies, NADPH oxidase (Nox) has been identified as a major source of ROS contributing to the development of AA (Miller et al., 2002; Yang et al., 2010; Jimenez-Altayo et al., 2018; van der Pluijm et al., 2018). Ablation of Nox1, Nox2, Nox4 isoforms or of the non-catalytic Nox subunit p47 in mice prevented the development of AAA (Lu et al., 2016; Siu et al., 2017). This is explained by the fact that Nox-derived ROS are important second messengers in the vasculature, however, excessive ROS lead to increase MMPs activation as well as inflammatory and apoptotic factors, which contribute to the pathogenesis of AAA (Finkel, 2011; Miki and Funato, 2012; Holmstrom and Finkel, 2014; Brown and Griendling, 2015; Forrester et al., 2018; Quintana and Taylor, 2019). Similarly, the expression of anti-oxidant enzymes such as superoxide dismutase, glutathione peroxidase, glutathione reductase, and glutathione S-transferase decreases in aortae of AAA and TAA patients (Dubick et al., 1999; Liao et al., 2008; Zuniga-Munoz et al., 2017).

Endothelial and VSM SirT1 are known to regulate the cellular redox state in the vascular wall by multiple mechanisms, including direct deacetylation of FOXOs, NF-kB, Nrf2, mitochondrial superoxide dismutase and Nox, which overall decrease ROS production (Fry et al., 2015; Huang et al., 2015; Zhang et al., 2017). Moreover, SirT1 inhibits the mitochondrial adaptor protein p66^Shc^, a critical modulator of intracellular redox state and a major contributor of oxidative damage-induced endothelial dysfunction in the settings of diabetes (Zhou et al., 2011; Paneni et al., 2013), by directly deacetylating its lysine 81 (Kumar et al., 2017). Taken together, these well-established anti-oxidant effects of SirT1 are consistent with the findings that VSM-specific SirT1 deletions in mice, and decreased expression of SirT1 in human aorta, are associated with aortic dissection or aneurysms (Fry et al., 2015; Chen et al., 2016).

Interestingly, SirT1 itself is a redox-sensitive enzyme. We have shown that SirT1 oxidative post-translational modifications, namely S-glutathionylation, at cysteine residues of its catalytic domain, profoundly affects its enzymatic activity (Zee et al., 2010; Shao et al., 2014). Reversible oxidative post-translational modifications, such as S-glutathionylation, result in activation or inactivation of proteins, thereby regulating signaling cascades and preventing irreversible oxidation of protein thiols in the settings of oxidative stress (Cohen et al., 2016; Byrne et al., 2020). Consistent with our findings, redox factor-1 and apurinic/apyrimidinic endonuclease-1, two cellular reducing agents, are able to restore NO bioavailability and endothelium-dependent vasorelaxation, through the reduction of conserved cysteine sulfhydryls in the SirT1 deacetylase domain (Jung et al., 2013). The loss of this fine-tuned mechanism in human endothelial cells exposed to oxidant stress, such as after exposure to cigarette smoke or hydrogen peroxide, have been associated with endothelial dysfunction (Chung et al., 2010). More recently, we found that H_2_O_2_, as well as TGF-β1, a pro-fibrotic cytokine known to be activated in Marfan’s syndrome and to upregulate Nox4 in the vasculature (Lu et al., 2016; Siu et al., 2017; Zuniga-Munoz et al., 2017; Jimenez-Altayo et al., 2018), increases the reversible oxidation of SirT1 in aortic VSM cells (E. Budbazar and F. Seta, unpublished results). Therefore, an impairment of SirT1 activity by oxidative post-translational modifications may be causally linked to the development of AA, at least in individuals with Marfan’s syndrome, possibly by exacerbating the deleterious effects of oxidant stress, whereas preventing or boosting SirT1 activity in the aortic wall may prevent AA.

## SirT1 and HO-1 in Aortic Aneurysm

Heme oxygenase-1 (HO-1) and 2 (HO-2) catalyze the rate-limiting step of the cellular degradation of heme. In the presence of oxygen and cofactors, HO-1 and HO-2 metabolize heme into carbon monoxide (CO), free iron and biliverdin, subsequently converted to bilirubin (BR; Abraham and Kappas, 2005). Both HO-1 and HO-2 isoforms are catalytically active in the vasculature (Thorup et al., 1999; Zhang et al., 2001), as demonstrated by HO-dependent release of CO in rat aorta, renal and cerebral arteries, and gracilis muscle arterioles (Kaide et al., 2001; Zhang et al., 2001).

HO-1 and HO-2 regulate vascular tone and hemodynamic function, mainly through CO production (Kaide et al., 2001, 2004; Zhang et al., 2001; Rodriguez et al., 2003; Arregui et al., 2004). We and others have shown that HO inhibition causes vasoconstriction (Kaide et al., 2001, 2004; Zhang et al., 2001; Rodriguez et al., 2004) while CO generally promotes vasodilation (Kaide et al., 2001, 2004; Arregui et al., 2004; Rodriguez et al., 2011). The vasoconstrictor effects associated with HO inhibitors are greater after NO synthesis inhibition (Zhang et al., 2001; Rodriguez et al., 2003), linked in part, to amplification of prevailing neurohormonal constrictor mechanisms (Rodriguez et al., 2003). Importantly, NO synthesis inhibition elevates CO formation both in vivo and in vitro (Rodriguez et al., 2004). Therefore, the significance of the HO–CO system might be particularly relevant in the settings of reduced NO bioavailability, as it is the case for conditions associated with increased oxidant stress and reduced NO levels, including numerous vascular and renal diseases (Salom et al., 2007; Rodriguez et al., 2011; Bonacasa et al., 2013).

HO-1 expression, but not HO-2, is increased in cultured VSM and endothelial cells in response to various stress stimuli (Christodoulides et al., 1995). Similar to SirT1, HO-1 overexpression serves a protective role by virtue of anti-oxidant (Ferrandiz and Devesa, 2008; Bonacasa et al., 2013), anti-inflammatory (Lee et al., 2004), anti-apoptotic (Ferrandiz and Devesa, 2008), and anti-proliferative (Deng et al., 2004; Lee et al., 2004) effects in endothelial, smooth muscle cells and macrophages in the vascular wall (Kim et al., 2011), by increasing CO and/or biliverdin production or by reducing the pro-oxidant heme levels (Abraham and Kappas, 2005; Duvigneau et al., 2019). Like SirT1, HO-1 confers protection in several vascular injury models, such us ischemic heart disease, atherosclerosis, hypertension, diabetes, or vascular proliferative diseases (Abraham and Kappas, 2005; Loboda et al., 2008; Kim et al., 2011). Interestingly, single nucleotide polymorphisms in the *HO-1* promoter region, which results in a decreased ability to upregulate HO-1, has been linked to AA disease risk in humans (Schillinger et al., 2002). HO-1 deficiency in mice exacerbates the development of AA (Azuma et al., 2016; Ho et al., 2016), whereas HO-1 overexpression, induced by heme treatment (Azuma et al., 2016) or shear stress (Nakahashi et al., 2002), attenuated AA progression by opposing inflammation (Azuma et al., 2016; Ho et al., 2016) and oxidative stress (Nakahashi et al., 2002; Ho et al., 2016).

Overall, HO-1 and SirT1 are part of the integrated system that modulates the cellular response to stress and might positively interact to regulate cardiovascular function. Treatment with cobalt protoporphyrin, a HO-1 chemical inducer (Sodhi et al., 2015), or HO-1 overexpression in macrophages (Nakamura et al., 2018), consistently enhanced SirT1 expression. Conversely, SirT1 activity can modulate HO-1 biological effects in hepatic cells (Sodhi et al., 2015; Nakamura et al., 2018), adypocites (Lakhani et al., 2019), or myocardial tissue (Yu et al., 2019). Moreover, in a human model of endogenous AT1R antagonism, SirT1 directly associates with improved NO-dependent vasodilation via HO-1 (Davis et al., 2013). These interactions suggest that positive feedback mechanisms between SirT1 and HO-1 might be at play in the vasculature, and they may affect HO-1-mediated cytoprotection in cardiovascular diseases. To this end, pharmacological activation of miR-181b/SirT1/HO-1 signaling axis reduced the development of ang II-induced AAA in apoE-/- mice (Hou et al., 2019). Although the exact molecular mechanisms accounting for the interaction between SirT-1 and HO-1, and its functional significance in vascular pathology, remain to be fully elucidated, the development of specific targeted therapies that modulate the SirT1/HO-1 axis could represent a new therapeutic strategy for the management of AA disease, as well as other vascular diseases.

## Sex Differences, Oxidative Stress, and Susceptibility to AA

Aortic aneurysm is more likely to occur in men than in women (Singh et al., 2001) but women have a greater risk of AA rupture and poorer outcome than men. Several factors may contribute to sex-specific susceptibility of male to AA namely differences in mechanical properties of the aorta, levels of aortic wall MMPs, renin angiotensin system activity, or inflammatory and immune responses, which might be influenced by sex hormones, as thoroughly reviewed elsewhere (Boese et al., 2018; Robinet et al., 2018). Interestingly, to dissociate the effects of sex hormones from sex chromosome, Alsiraj et al. (2017) used the four core genotype mouse model to create XX and XY female offspring, showing that XY phenotypic females had increased AA incidence and severity compared with XX females, through increased inflammation and oxidative stress. Moreover, castration prevented the progression of disease in a model of angII-induced AA (Zhang et al., 2015). Since estrogen and testosterone also differently influence redox balance (Boese et al., 2018), the higher susceptibility of male to oxidative stress and AA in animal studies, might be related to either the effects of sex hormones or sex chromosomes genes on oxidant pathways.

Increased activation of Nox and ROS production are known to be higher (Dantas et al., 2004), while the antioxidant capacity is decreased (Rodriguez et al., 2010; Bonacasa et al., 2013), in males compared to females in preclinical and clinical studies (Sartori-Valinotti et al., 2007). Estrogen increases HO-1 expression in cultured endothelial cells (Baruscotti et al., 2010; Marcantoni et al., 2012) and the uterus of ovariectomized animals (Cella et al., 2006). In contrast, HO-1 expression is higher in females compared to males in several experimental models of cardiovascular disease (Zampino et al., 2006; Bonacasa et al., 2013). Noteworthy, our own work and others’, showed that increased HO-1 expression and/or activity in female rats was associated with decreased nitrosative stress and glomerular damage in the diabetic kidney (Bonacasa et al., 2013) and a lower susceptibility to cardiac ischemia compared to males (Posa et al., 2013). In line with this notion, increased HO-1 expression and activity in the female offspring of diabetic mothers, was associated with lower nitrotyrosine levels, and improved cardiovascular function in females compared to males (F. Rodriguez and J. M. Sanchez, unpublished results). Therefore, antioxidant HO-1 activity could improve vascular function partly by compensating for the loss of NO bioavailability, in vascular diseases, particularly in females.

Similarly to HO-1, SirT1 activity may be influenced by sex or sex hormones. Estrogen induces SirT1 and SirT1/AMPK/histone H3 pathway, which relates to the cardiovascular protective effects of estrogen therapy (Bendale et al., 2013). Moreover, SirT-1 downregulation in the female, but not male, hearts directly correlated with a decline in mitochondrial anti-oxidative defense and a pro-inflammatory shift (Barcena de Arellano et al., 2019). Consistently, sex-specific downregulation of the SirT1/AMPK/FOXO3a/PGC-1α regulatory network was observed in male, but not female offspring, in response to maternal high-fat feeding (Nguyen et al., 2017). Overall, the possibility of a sex-specific regulation of SirT1 and HO-1 in the context of oxidative stress, such as in AA, is intriguing. Further studies addressing the functional significance of sex specific changes in SirT1 and HO-1 in AA, and the correlation with clinical features are warranted.

## Translational Therapeutic Applications of SirT1 or HO Activation for AA

**FIGURE 1:**
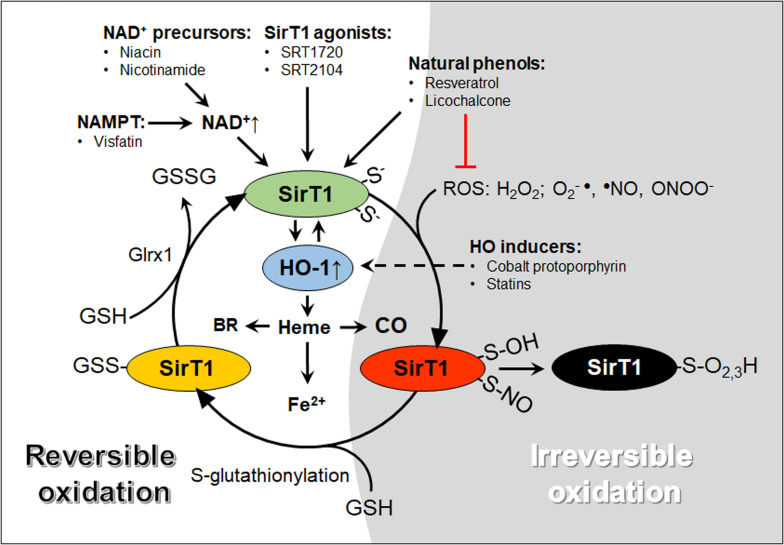
A graphical abstract of oxidative modifications of cysteine thiols and pharmacological agents that increase SirT1 and HO-1 activity. The reactive thiolate anions of SirT1 exposed to ROS can react to form reversible (red and yellow, temporary inactivation) or irreversible (black, permanent inactivation). Glutathione (GSH) adducts added by S-glutathionylation inactivate SirT1. SirT1-GSH adducts may be reversed by glutaredoxin-1 (Glrx1), forming oxidized GSH (GSSG) and active SirT1 (green). HO-1 may interact with SirT1 through positive feedback mechanisms and metabolize heme into carbon monoxide (CO), free iron (Fe^2+^) and biliverdin, subsequently converted to bilirubin (BR). NAD^+^, nicotinamide adenine dinucleotide; NAMPT, nicotinamide phosphoribosyltransferase; ROS, reactive oxygen species.

Substantial R&D has been invested in the quest of compounds that could boost SirT1 activity for the treatment of cardiovascular diseases. Thus far, numerous plant-derived molecules have shown promising results, although proving their pharmacological specificity remains a challenge. Liquorice-derived licochalcone attenuates angII-induced AAA by modulating the miR-181b/SirT1/HO-1 signaling axis (Hou et al., 2019). Resveratrol, a grape-derived non-toxic polyphenol known to activate SirT1, decreases the incidence of AAA in angII-infused apoE^–/–^ mice fed a high fat diet by increasing ACE2 and downregulating NF-kB, Akt, ERK1/2 and ATR1 in human aortic VSM (Moran et al., 2017). Similarly, resveratrol, but not another putative SirT1 agonist SRT1720, prevented the development of TAA in Fbn1^C1041G/+^ mice, a model of Marfan’s syndrome, by stimulating an endothelial cell-derived factor, which downregulated miR-29b and upregulated Bcl-2 in VSM cells, inhibiting their apoptosis (Hibender et al., 2016). However, the beneficial effects of resveratrol and other putative SirT1-specific agonists, such as SRT1720 and SRT2104, are attributed to both SirT1-dependent and -independent mechanisms (Park et al., 2012; Zordoky et al., 2015; Bonnefont-Rousselot, 2016; Hibender et al., 2016; Moran et al., 2017; van Andel et al., 2019). It is well established that resveratrol can activate AMPK, inhibit cyclooxygenases and affect a variety of other enzymes (Bonnefont-Rousselot, 2016; Wicinski et al., 2018). Likewise, novel SirT1 activators, which were effective in decreasing arterial stiffness and improving the lipid profile of healthy cigarette smokers and elderly volunteers in clinical trials (Libri et al., 2012; Venkatasubramanian et al., 2013, 2016), function as partial agonists of SirT1 or exhibit off-target effects (Pacholec et al., 2010). Therefore, attempts to develop alternative approaches to activate SirT1, rather than direct agonists, are an active field of research. Manipulating the metabolism of NAD^+^, the essential co-factor for SirT1 deacetylase activity, has proven very promising, as in some cases, it recapitulates the effects of resveratrol (Baur, 2010). Both niacin and nicotinamide supplementation increases NAD^+^ levels and NAD^+^-dependent SirT1 activity in aortas and prevents the development of AAA in mice (Horimatsu et al., 2019). Similarly, nicotinamide phosphoribosyltransferase (NAMPT), also known as visfatin, a methyltransferase crucial for the synthesis of the NAD^+^ precursor nicotinamide mononucleotide (Galli et al., 2013), has been shown to maintain aortic VSM integrity through NAD/SirT1 pathway (Watson et al., 2017). Additionally, we propose that understanding the role of oxidative post-translational modifications, such as S-glutathionylation of SirT1 cysteine residues, may lead to novel therapeutic strategies (Figure 1). We previously shown that a redox-resistant mutant SirT1 and overexpression of glutaredoxin-1, a thioltransferase that removes S-glutathionylation on SirT1, protects against liver metabolic disease (Shao et al., 2014, 2017). Whether a similar approach could be implemented in the clinic to treat AA and other cardiovascular diseases warrants further research.

Lipid-lowering agents widely used in clinical settings, such as probucol or statins, showed anti-proliferative effects on VSM through HO-1 induction (Deng et al., 2004; Lee et al., 2004), and decreased the development of experimental AA by inducing HO-1 gene expression (Azuma et al., 2016; Piechota-Polanczyk et al., 2018; Chen et al., 2020). Likewise, simvastatin-treated patients with AA, showed higher levels of anti-oxidant HO-1 in vascular tissue (Piechota-Polanczyk et al., 2018). Taken together, these findings strongly support a key protective role of HO-1 in limiting AA progression, and that statins and probucol may limit AA progression through mechanisms involving HO-1, probably independently of their lipid-lowering effects.

In contrast, it is important to consider that deleterious effects of HO-1 (Jais et al., 2014) and SirT1 overexpression (Kawashima et al., 2011) have been reported. In the case of HO-1 induction, these deleterious effects relate to increased reactive free iron (Suttner and Dennery, 1999) or reduced heme availability (Duvigneau et al., 2019). Interestingly, it has been suggested that there is a beneficial threshold of HO-1 overexpression (Suttner and Dennery, 1999; Namba et al., 2014) operating in specific subcellular localizations (Namba et al., 2014). Therefore, attempts at targeting HO-1 enzymatic activity to treat AA, as well as other vascular diseases, should aim at generating sufficient amounts of HO-1 activity, in specific cell compartments, maintaining suitable levels of intracellular heme and heme derived products formed by HO, avoiding any undesirable effects. Similarly, SirT1 activation or overexpression should be titrated to avoid unwanted effects.

Overall, HO-1 and SirT1 are part of gene complexes termed *vitagenes* (Calabrese et al., 2014), which provide an integrated response to control oxidant stress-induced tissue injury. Vitagenes products (i.e., heat shock proteins, BR, CO, gluthathione) mediate anti-oxidant, anti-apoptotic, anti-proliferative and anti-inflammatory actions (Abraham and Kappas, 2005; Calabrese et al., 2014). Dietary anti-oxidants (Abraham and Kappas, 2005; Moran et al., 2017; Hou et al., 2019) and clinically widely used compounds, such us statins, have shown favorable pleiotropic effects partly mediated by HO-1 induction (Lee et al., 2004; Piechota-Polanczyk et al., 2018) or SirT1 expression (Strycharz et al., 2018), providing a strong rationale for their therapeutic benefits in cardiovascular diseases, including AA (Hou et al., 2019). Therefore, the importance of developing pharmacological agents that activate an integrated response against oxidant stress in AA seems evident. Still, there is no definitive evidence from large scale clinical studies with anti-oxidant supplementation (Egea et al., 2017), requiring a better understanding of how each compound would affect the sources of ROS in specific cell compartments, modulate NAD + levels (Horimatsu et al., 2019), or affect ROS-induced reversible modifications, such as protein S-glutathionylation (Shao et al., 2014, 2017), for the suitable translation into clinical settings.

## Author Contributions

EB wrote and reviewed the manuscript, and prepared the figure. FR and FS wrote and reviewed the manuscript. JS reviewed the manuscript. All authors contributed to the article and approved the submitted version.

## Conflict of Interest

The authors declare that the research was conducted in the absence of any commercial or financial relationships that could be construed as a potential conflict of interest.

## References

[B1] Abraham N. G. Kappas A. (2005). Heme oxygenase and the cardiovascular-renal system. *Free Radic. Biol. Med*. 39 1–25. 10.1016/j.freeradbiomed.2005.03.010 15925276

[B2] Alsiraj Y. Thatcher S. E. Charnigo R. Chen K. Blalock E. Daugherty A. (2017). Female mice with an xy sex chromosome complement develop severe angiotensin ii-induced abdominal aortic aneurysms. *Circulation* 135 379–391. 10.1161/circulationaha.116.023789 27815372 PMC5470072

[B3] Arregui B. Lopez B. Garcia Salom M. Valero F. Navarro C. Fenoy F. J. (2004). Acute renal hemodynamic effects of dimanganese decacarbonyl and cobalt protoporphyrin. *Kidney Int.* 65 564–574. 10.1111/j.1523-1755.2004.00409.x 14717926

[B4] Azuma J. Wong R. J. Morisawa T. Hsu M. Maegdefessel L. Zhao H. (2016). Heme oxygenase-1 expression affects murine abdominal aortic aneurysm progression. *PLoS One* 11:e0149288. 10.1371/journal.pone.0149288 26894432 PMC4760983

[B5] Barcena de Arellano M. L. Pozdniakova S. Kuhl A. A. Baczko I. Ladilov Y. Regitz-Zagrosek V. (2019). Sex differences in the aging human heart: decreased sirtuins, pro-inflammatory shift and reduced anti-oxidative defense. *Aging* 11 1918–1933. 10.18632/aging.101881 30964749 PMC6503880

[B6] Baruscotti I. Barchiesi F. Jackson E. K. Imthurn B. Stiller R. Kim J. H. (2010). Estradiol stimulates capillary formation by human endothelial progenitor cells: role of estrogen receptor-{alpha}/{beta}, heme oxygenase 1, and tyrosine kinase. *Hypertension* 56 397–404. 10.1161/hypertensionaha.110.153262 20644008 PMC2936691

[B7] Baur J. A. (2010). Biochemical effects of sirt1 activators. *Biochim. Biophys. Acta* 1804 1626–1634. 10.1016/j.bbapap.2009.10.025 19897059 PMC2886178

[B8] Bendale D. S. Karpe P. A. Chhabra R. Shete S. P. Shah H. Tikoo K. (2013). 17-beta oestradiol prevents cardiovascular dysfunction in post-menopausal metabolic syndrome by affecting sirt1/ampk/h3 acetylation. *Br. J. Pharmacol.* 170 779–795. 10.1111/bph.12290 23826814 PMC3799593

[B9] Boese A. C. Chang L. Yin K. J. Chen Y. E. Lee J. P. Hamblin M. H. (2018). Sex differences in abdominal aortic aneurysms. *Am. J. Physiol. Heart Circ. Physiol*. 314 H1137–H1152. 10.1152/ajpheart.00519.2017 29350999 PMC6032079

[B10] Bonacasa B. Perez C. Salom M. G. Lopez B. Saez-Belmonte F. Martinez P. (2013). Sexual dimorphism in renal heme-heme oxygenase system in the streptozotocin diabetic rats. *Curr. Pharm. Des*. 19 2678–2686. 10.2174/1381612811319150002 23092315

[B11] Bonnefont-Rousselot D. (2016). Resveratrol and cardiovascular diseases. *Nutrients* 8:250. 10.3390/nu8050250 27144581 PMC4882663

[B12] Brown D. I. Griendling K. K. (2015). Regulation of signal transduction by reactive oxygen species in the cardiovascular system. *Circ. Res*. 116 531–549. 10.1161/circresaha.116.303584 25634975 PMC4392388

[B13] Brunet A. Sweeney L. B. Sturgill J. F. Chua K. F. Greer P. L. Lin Y. (2004). Stress-dependent regulation of foxo transcription factors by the sirt1 deacetylase. *Science* 303 2011–2015. 10.1126/science.1094637 14976264

[B56] Byrne D. P. Shrestha S. Galler M. Cao M. Daly L. A. Campbell A. E. (2020). Aurora A regulation by reversible cysteine oxidation reveals evolutionarily conserved redox control of Ser/Thr protein kinase activity. *Sci. Signal* 13:eaax2713 10.1126/scisignal.aax2713 32636306

[B14] Calabrese V. Scapagnini G. Davinelli S. Koverech G. Koverech A. De Pasquale C. (2014). Sex hormonal regulation and hormesis in aging and longevity: role of vitagenes. *J. Cell Commun. Signal*. 8 369–384. 10.1007/s12079-014-0253-7 25381162 PMC4390801

[B15] Cella M. Farina M. G. Keller Sarmiento M. I. Chianelli M. Rosenstein R. E. Franchi A. M. (2006). Heme oxygenase-carbon monoxide (ho-co) system in rat uterus: effect of sexual steroids and prostaglandins. *J. Steroid Biochem. Mol. Biol*. 99 59–66. 10.1016/j.jsbmb.2005.11.007 16524721

[B16] Chen C. Wang Y. Cao Y. Wang Q. Anwaier G. Zhang Q. (2020). Mechanisms underlying the inhibitory effects of probucol on elastase-induced abdominal aortic aneurysm in mice. *Br. J. Pharmacol*. 177 204–216. 10.1111/bph.14857 31478560 PMC6976779

[B17] Chen H. Z. Wang F. Gao P. Pei J. F. Liu Y. Xu T. T. (2016). Age-associated sirtuin 1 reduction in vascular smooth muscle links vascular senescence and inflammation to abdominal aortic aneurysm. *Circ. Res*. 119 1076–1088. 10.1161/circresaha.116.308895 27650558 PMC6546422

[B18] Christodoulides N. Durante W. Kroll M. H. Schafer A. I. (1995). Vascular smooth muscle cell heme oxygenases generate guanylyl cyclase-stimulatory carbon monoxide. *Circulation* 91 2306–2309. 10.1161/01.cir.91.9.2306 7729015

[B19] Chung S. Yao H. Caito S. Hwang J. W. Arunachalam G. Rahman I. (2010). Regulation of sirt1 in cellular functions: role of polyphenols. *Arch. Biochem. Biophys*. 501 79–90. 10.1016/j.abb.2010.05.003 20450879 PMC2930135

[B20] Cohen R. A. Murdoch C. E. Watanabe Y. Bolotina V. M. Evangelista A. M. Haeussler D. J. (2016). Endothelial cell redox regulation of ischemic angiogenesis. *J. Cardiovasc. Pharmacol*. 67 458–464. 10.1097/fjc.0000000000000381 26927696 PMC4899292

[B21] Dantas A. P. Franco Mdo C. Silva-Antonialli M. M. Tostes R. C. Fortes Z. B. Nigro D. (2004). Gender differences in superoxide generation in microvessels of hypertensive rats: role of nad(p)h-oxidase. *Cardiovasc. Res*. 61 22–29. 10.1016/j.cardiores.2003.10.010 14732198

[B22] Davies M. J. (1998). Aortic aneurysm formation: lessons from human studies and experimental models. *Circulation* 98 193–195. 10.1161/01.cir.98.3.193 9697816

[B23] Davis P. A. Pagnin E. Dal Maso L. Caielli P. Maiolino G. Fusaro M. (2013). Sirt1, heme oxygenase-1 and no-mediated vasodilation in a human model of endogenous angiotensin ii type 1 receptor antagonism: implications for hypertension. *Hypertens. Res.* 36 873–878. 10.1038/hr.2013.48 23698802

[B24] Deng Y. M. Wu B. J. Witting P. K. Stocker R. (2004). Probucol protects against smooth muscle cell proliferation by upregulating heme oxygenase-1. *Circulation* 110 1855–1860. 10.1161/01.cir.0000142610.10530.25 15364806

[B25] D’Onofrio N. Servillo L. Balestrieri M. L. (2018). Sirt1 and sirt6 signaling pathways in cardiovascular disease protection. *Antioxid. Redox Signal*. 28 711–732. 10.1089/ars.2017.7178 28661724 PMC5824538

[B26] Dubick M. A. Keen C. L. DiSilvestro R. A. Eskelson C. D. Ireton J. Hunter G. C. (1999). Antioxidant enzyme activity in human abdominal aortic aneurysmal and occlusive disease. *Proc. Soc. Exp. Biol. Med. Soc. Exp. Biol. Med*. 220 39–45. 10.1046/j.1525-1373.1999.d01-6.x 9893167

[B27] Duvigneau J. C. Esterbauer H. Kozlov A. V. (2019). Role of heme oxygenase as a modulator of heme-mediated pathways. *Antioxidants* 8:475. 10.3390/antiox8100475 31614577 PMC6827082

[B28] Egea J. Fabregat I. Frapart Y. M. Ghezzi P. Gorlach A. Kietzmann T. (2017). European contribution to the study of ros: a summary of the findings and prospects for the future from the cost action bm1203 (eu-ros). *Redox Biol*. 13 94–162. 28577489 10.1016/j.redox.2017.05.007 PMC5458069

[B29] Ferrandiz M. L. Devesa I. (2008). Inducers of heme oxygenase-1. *Curr. Pharm. Des*. 14 473–486. 10.2174/138161208783597399 18289074

[B30] Finkel T. (2011). Signal transduction by reactive oxygen species. *J. Cell Biol*. 194 7–15. 10.1083/jcb.201102095 21746850 PMC3135394

[B31] Forrester S. J. Kikuchi D. S. Hernandes M. S. Xu Q. Griendling K. K. (2018). Reactive oxygen species in metabolic and inflammatory signaling. *Circ. Res*. 122 877–902. 10.1161/circresaha.117.311401 29700084 PMC5926825

[B32] Fry J. L. Al Sayah L. Weisbrod R. M. Van Roy I. Weng X. Cohen R. A. (2016). Vascular smooth muscle sirtuin-1 protects against diet-induced aortic stiffness. *Hypertension* 68 775–784. 10.1161/hypertensionaha.116.07622 27432859 PMC4982825

[B33] Fry J. L. Shiraishi Y. Turcotte R. Yu X. Gao Y. Z. Akiki R. (2015). Vascular smooth muscle sirtuin-1 protects against aortic dissection during angiotensin ii-induced hypertension. *J. Am. Heart Assoc*. 4:e002384. 10.1161/JAHA.115.002384 26376991 PMC4599512

[B34] Frye R. A. (2000). Phylogenetic classification of prokaryotic and eukaryotic sir2-like proteins. *Biochem. Biophys. Res. Commun*. 273 793–798. 10.1006/bbrc.2000.3000 10873683

[B35] Galatioto J. Caescu C. I. Hansen J. Cook J. R. Miramontes I. Iyengar R. (2018). Cell type-specific contributions of the angiotensin ii type 1a receptor to aorta homeostasis and aneurysmal disease-brief report. *Arterioscler. Thromb. Vasc. Biol*. 38 588–591. 10.1161/atvbaha.117.310609 29371244 PMC5823778

[B36] Galli U. Travelli C. Massarotti A. Fakhfouri G. Rahimian R. Tron G. C. (2013). Medicinal chemistry of nicotinamide phosphoribosyltransferase (nampt) inhibitors. *J. Med. Chem*. 56 6279–6296. 10.1021/jm4001049 23679915

[B37] Gomez D. Kessler K. Michel J. B. Vranckx R. (2013). Modifications of chromatin dynamics control smad2 pathway activation in aneurysmal smooth muscle cells. *Circ. Res*. 113 881–890. 10.1161/circresaha.113.301989 23825360

[B38] Gorenne I. Kumar S. Gray K. Figg N. Yu H. Mercer J. (2013). Vascular smooth muscle cell sirtuin 1 protects against DNA damage and inhibits atherosclerosis. *Circulation* 127 386–396. 10.1161/circulationaha.112.124404 23224247

[B39] Guarente L. (2013). Calorie restriction and sirtuins revisited. *Genes Dev*. 27 2072–2085. 10.1101/gad.227439.113 24115767 PMC3850092

[B40] Haigis M. C. Sinclair D. A. (2010). Mammalian sirtuins: biological insights and disease relevance. *Ann. Rev. Pathol*. 5 253–295. 10.1146/annurev.pathol.4.110807.092250 20078221 PMC2866163

[B41] Hibender S. Franken R. van Roomen C. Ter Braake A. van der Made I. Schermer E. E. (2016). Resveratrol inhibits aortic root dilatation in the fbn1c1039g/+ marfan mouse model. *Arterioscler. Thromb. Vasc. Biol*. 36 1618–1626. 10.1161/atvbaha.116.307841 27283746 PMC4961273

[B42] Ho Y. C. Wu M. L. Gung P. Y. Chen C. H. Kuo C. C. Yet S. F. (2016). Heme oxygenase-1 deficiency exacerbates angiotensin ii-induced aortic aneurysm in mice. *Oncotarget* 7 67760–67776. 10.18632/oncotarget.11917 27626316 PMC5356517

[B43] Holmstrom K. M. Finkel T. (2014). Cellular mechanisms and physiological consequences of redox-dependent signalling. *Nat. Rev. Mol. Cell Biol*. 15 411–421. 10.1038/nrm3801 24854789

[B44] Horimatsu T. Blomkalns A. L. Ogbi M. Moses M. Kim D. Patel S. (2019). Niacin protects against abdominal aortic aneurysm formation via gpr109a independent mechanisms: role of nad+/nicotinamide. *Cardiovasc. Res.* 10.1093/cvr/cvz303 [Online ahead of print] 31710686 PMC7695356

[B45] Hou X. Yang S. Zheng Y. (2019). Licochalcone a attenuates abdominal aortic aneurysm induced by angiotensin ii via regulating the mir-181b/sirt1/ho-1 signaling. *J. Cell. Physiol*. 234 7560–7568. 10.1002/jcp.27517 30417353

[B46] Huang K. Yan Z. Q. Zhao D. Chen S. G. Gao L. Z. Zhang P. (2015). Sirt1 and foxo mediate contractile differentiation of vascular smooth muscle cells under cyclic stretch. *Cell. Physiol. Biochem.* 37 1817–1829. 10.1159/000438544 26584282

[B47] Jais A. Einwallner E. Sharif O. Gossens K. Lu T. T. Soyal S. M. (2014). Heme oxygenase-1 drives metaflammation and insulin resistance in mouse and man. *Cell* 158 25–40. 10.1016/j.cell.2014.04.043 24995976 PMC5749244

[B48] Jeong J. Juhn K. Lee H. Kim S. H. Min B. H. Lee K. M. (2007). Sirt1 promotes DNA repair activity and deacetylation of ku70. *Exp. Mol. Med*. 39 8–13. 10.1038/emm.2007.2 17334224

[B49] Jimenez-Altayo F. Meirelles T. Crosas-Molist E. Sorolla M. A. Del Blanco D. G. Lopez-Luque J. (2018). Redox stress in marfan syndrome: dissecting the role of the nadph oxidase nox4 in aortic aneurysm. *Free Radic. Biol. Med*. 118 44–58. 29471108 10.1016/j.freeradbiomed.2018.02.023

[B50] Jones A. Deb R. Torsney E. Howe F. Dunkley M. Gnaneswaran Y. (2009). Rosiglitazone reduces the development and rupture of experimental aortic aneurysms. *Circulation* 119 3125–3132. 10.1161/circulationaha.109.852467 19506106

[B51] Jung S. B. Kim C. S. Kim Y. R. Naqvi A. Yamamori T. Kumar S. (2013). Redox factor-1 activates endothelial sirtuin1 through reduction of conserved cysteine sulfhydryls in its deacetylase domain. *PLoS One* 8:e65415. 10.1371/journal.pone.0065415 23755229 PMC3670896

[B52] Kaide J. Zhang F. Wei Y. Wang W. Gopal V. R. Falck J. R. (2004). Vascular co counterbalances the sensitizing influence of 20-hete on agonist-induced vasoconstriction. *Hypertension* 44 210–216. 10.1161/01.hyp.0000135658.57547.bb 15226275

[B53] Kaide J. I. Zhang F. Wei Y. Jiang H. Yu C. Wang W. H. (2001). Carbon monoxide of vascular origin attenuates the sensitivity of renal arterial vessels to vasoconstrictors. *J. Clin. Invest*. 107 1163–1171. 10.1172/jci11218 11342580 PMC209275

[B54] Kawai Y. Garduno L. Theodore M. Yang J. Arinze I. J. (2011). Acetylation-deacetylation of the transcription factor nrf2 (nuclear factor erythroid 2-related factor 2) regulates its transcriptional activity and nucleocytoplasmic localization. *J. Biol. Chem*. 286 7629–7640. 10.1074/jbc.m110.208173 21196497 PMC3045017

[B55] Kawashima T. Inuzuka Y. Okuda J. Kato T. Niizuma S. Tamaki Y. (2011). Constitutive sirt1 overexpression impairs mitochondria and reduces cardiac function in mice. *J. Mol. Cell Cardiol*. 51 1026–1036. 10.1016/j.yjmcc.2011.09.013 21964378

[B57] Kim Y. M. Pae H. O. Park J. E. Lee Y. C. Woo J. M. Kim N. H. (2011). Heme oxygenase in the regulation of vascular biology: from molecular mechanisms to therapeutic opportunities. *Antioxid. Redox Signal*. 14 137–167. 10.1089/ars.2010.3153 20624029 PMC2988629

[B58] Kumar S. Kim Y. R. Vikram A. Naqvi A. Li Q. Kassan M. (2017). Sirtuin1-regulated lysine acetylation of p66shc governs diabetes-induced vascular oxidative stress and endothelial dysfunction. *Proc. Natl. Acad. Sci. U.S.A*. 114 1714–1719. 10.1073/pnas.1614112114 28137876 PMC5321021

[B59] Lakhani H. V. Zehra M. Pillai S. S. Puri N. Shapiro J. I. Abraham N. G. (2019). Beneficial role of ho-1-sirt1 axis in attenuating angiotensin ii-induced adipocyte dysfunction. *Int. J. Mol. Sci.* 20:3205. 10.3390/ijms20133205 31261892 PMC6650875

[B60] Lee T. S. Chang C. C. Zhu Y. Shyy J. Y. (2004). Simvastatin induces heme oxygenase-1: a novel mechanism of vessel protection. *Circulation* 110 1296–1302. 10.1161/01.cir.0000140694.67251.9c 15337692

[B61] Leeper N. J. Raiesdana A. Kojima Y. Kundu R. K. Cheng H. Maegdefessel L. (2013). Loss of cdkn2b promotes p53-dependent smooth muscle cell apoptosis and aneurysm formation. *Arterioscler. Thromb. Vasc. Biol*. 33 e1–e10. 23162013 10.1161/ATVBAHA.112.300399 PMC3569043

[B62] Li L. Zhang H. N. Chen H. Z. Gao P. Zhu L. H. Li H. L. (2011). Sirt1 acts as a modulator of neointima formation following vascular injury in mice. *Circ. Res*. 108 1180–1189. 10.1161/circresaha.110.237875 21474819

[B63] Liao M. Liu Z. Bao J. Zhao Z. Hu J. Feng X. (2008). A proteomic study of the aortic media in human thoracic aortic dissection: implication for oxidative stress. *J. Thoracic Cardiovasc. Surg.* 136 65–72. 10.1016/j.jtcvs.2007.11.017 18603055

[B64] Libri V. Brown A. P. Gambarota G. Haddad J. Shields G. S. Dawes H. (2012). A pilot randomized, placebo controlled, double blind phase i trial of the novel sirt1 activator srt2104 in elderly volunteers. *PLoS One* 7:e51395. 10.1371/journal.pone.0051395 23284689 PMC3527451

[B65] Ling L. Gu S. Cheng Y. (2017). Resveratrol inhibits adventitial fibroblast proliferation and induces cell apoptosis through the sirt1 pathway. *Mol. Med. Rep*. 15 567–572. 10.3892/mmr.2016.6098 28101569 PMC5364863

[B66] Liu Y. Wang T. T. Zhang R. Fu W. Y. Wang X. Wang F. (2016). Calorie restriction protects against experimental abdominal aortic aneurysms in mice. *J. Exp. Med*. 213 2473–2488. 10.1084/jem.20151794 27670594 PMC5068228

[B67] Loboda A. Jazwa A. Grochot-Przeczek A. Rutkowski A. J. Cisowski J. Agarwal A. (2008). Heme oxygenase-1 and the vascular bed: from molecular mechanisms to therapeutic opportunities. *Antioxid. Redox Signal*. 10 1767–1812. 10.1089/ars.2008.2043 18576916

[B68] Lu C. L. Liao M. T. Hou Y. C. Fang Y. W. Zheng C. M. Liu W. C. (2020). Sirtuin-1 and its relevance in vascular calcification. *Int. J. Mol. Sci.* 21:1593. 10.3390/ijms21051593 32111067 PMC7084838

[B69] Lu W. W. Jia L. X. Ni X. Q. Zhao L. Chang J. R. Zhang J. S. (2016). Intermedin1-53 attenuates abdominal aortic aneurysm by inhibiting oxidative stress. *Arterioscler. Thromb. Vasc. Biol*. 36 2176–2190. 10.1161/atvbaha.116.307825 27634835

[B70] Man A. W. C. Li H. Xia N. (2019). The role of sirtuin1 in regulating endothelial function, arterial remodeling and vascular aging. *Front. Physiol*. 10:1173. 10.3389/fphys.2019.01173 31572218 PMC6751260

[B71] Marampon F. Gravina G. L. Scarsella L. Festuccia C. Lovat F. Ciccarelli C. (2013). Angiotensin-converting-enzyme inhibition counteracts angiotensin ii-mediated endothelial cell dysfunction by modulating the p38/sirt1 axis. *J. Hypertens*. 31 1972–1983. 10.1097/hjh.0b013e3283638b32 23868084

[B72] Marcantoni E. Di Francesco L. Totani L. Piccoli A. Evangelista V. Tacconelli S. (2012). Effects of estrogen on endothelial prostanoid production and cyclooxygenase-2 and heme oxygenase-1 expression. *Prostaglandins Other Lipid Mediat*. 98 122–128. 10.1016/j.prostaglandins.2012.01.006 22330859

[B73] Mattagajasingh I. Kim C. S. Naqvi A. Yamamori T. Hoffman T. A. Jung S. B. (2007). Sirt1 promotes endothelium-dependent vascular relaxation by activating endothelial nitric oxide synthase. *Proc. Natl. Acad. Sci. U.S.A*. 104 14855–14860. 10.1073/pnas.0704329104 17785417 PMC1976244

[B74] Michishita E. Park J. Y. Burneskis J. M. Barrett J. C. Horikawa I. (2005). Evolutionarily conserved and nonconserved cellular localizations and functions of human sirt proteins. *Mol. Biol. Cell* 16 4623–4635. 10.1091/mbc.e05-01-0033 16079181 PMC1237069

[B75] Miki H. Funato Y. (2012). Regulation of intracellular signalling through cysteine oxidation by reactive oxygen species. *J. Biochem*. 151 255–261. 10.1093/jb/mvs006 22287686

[B76] Miller F. J. Jr. Sharp W. J. Fang X. Oberley L. W. Oberley T. D. Weintraub N. L. (2002). Oxidative stress in human abdominal aortic aneurysms: a potential mediator of aneurysmal remodeling. *Arterioscler. Thromb. Vasc. Biol*. 22 560–565. 10.1161/01.atv.0000013778.72404.30 11950691

[B77] Miyazaki R. Ichiki T. Hashimoto T. Inanaga K. Imayama I. Sadoshima J. (2008). Sirt1, a longevity gene, downregulates angiotensin ii type 1 receptor expression in vascular smooth muscle cells. *Arterioscler. Thromb. Vasc. Biol*. 28 1263–1269. 10.1161/atvbaha.108.166991 18420994

[B78] Mody P. S. Wang Y. Geirsson A. Kim N. Desai M. M. Gupta A. (2014). Trends in aortic dissection hospitalizations, interventions, and outcomes among medicare beneficiaries in the united states, 2000-2011. *Circulation* 7 920–928. 10.1161/circoutcomes.114.001140 25336626 PMC4380171

[B79] Moran C. S. Biros E. Krishna S. M. Wang Y. Tikellis C. Morton S. K. (2017). Resveratrol inhibits growth of experimental abdominal aortic aneurysm associated with upregulation of angiotensin-converting enzyme 2. *Arterioscler. Thromb. Vasc. Biol*. 37 2195–2203. 10.1161/atvbaha.117.310129 28935757

[B80] Motoki T. Kurobe H. Hirata Y. Nakayama T. Kinoshita H. Rocco K. A. (2015). Ppar-gamma agonist attenuates inflammation in aortic aneurysm patients. *Gen. Thoracic Cardiovasc. Surg*. 63 565–571. 10.1007/s11748-015-0576-1 26213347

[B81] Nakahashi T. K. Hoshina K. Tsao P. S. Sho E. Sho M. Karwowski J. K. (2002). Flow loading induces macrophage antioxidative gene expression in experimental aneurysms. *Arterioscler. Thromb. Vasc. Biol*. 22 2017–2022. 10.1161/01.atv.0000042082.38014.ea 12482828

[B82] Nakamura K. Kageyama S. Yue S. Huang J. Fujii T. Ke B. (2018). Heme oxygenase-1 regulates sirtuin-1-autophagy pathway in liver transplantation: from mouse to human. *Am. J. Transplant*. 18 1110–1121. 10.1111/ajt.14586 29136322 PMC5910267

[B83] Namba F. Go H. Murphy J. A. La P. Yang G. Sengupta S. (2014). Expression level and subcellular localization of heme oxygenase-1 modulates its cytoprotective properties in response to lung injury: a mouse model. *PLoS One* 9:e90936. 10.1371/journal.pone.0090936 24599172 PMC3944979

[B84] Nguyen L. T. Chen H. Pollock C. Saad S. (2017). SIRT1 reduction is associated with sex-specific dysregulation of renal lipid metabolism and stress responses in offspring by maternal high-fat diet. *Sci. Rep.* 7:8982. 10.1038/s41598-017-08694-4 28827681 PMC5567163

[B85] Oellerich M. F. Potente M. (2012). Foxos and sirtuins in vascular growth, maintenance, and aging. *Circ. Res*. 110 1238–1251. 10.1161/circresaha.111.246488 22539757

[B86] Ota H. Eto M. Kano M. R. Kahyo T. Setou M. Ogawa S. (2010a). Induction of endothelial nitric oxide synthase, sirt1, and catalase by statins inhibits endothelial senescence through the akt pathway. *Arterioscler. Thromb. Vasc. Biol*. 30 2205–2211. 10.1161/atvbaha.110.210500 20705918

[B87] Ota H. Eto M. Kano M. R. Ogawa S. Iijima K. Akishita M. (2008). Cilostazol inhibits oxidative stress-induced premature senescence via upregulation of sirt1 in human endothelial cells. *Arterioscler. Thromb. Vasc. Biol*. 28 1634–1639. 10.1161/atvbaha.108.164368 18556572

[B88] Ota H. Eto M. Ogawa S. Iijima K. Akishita M. Ouchi Y. (2010b). Sirt1/enos axis as a potential target against vascular senescence, dysfunction and atherosclerosis. *J. Atheroscler. Thromb*. 17 431–435. 10.5551/jat.3525 20215708

[B89] Pacholec M. Bleasdale J. E. Chrunyk B. Cunningham D. Flynn D. Garofalo R. S. (2010). Srt1720, srt2183, srt1460, and resveratrol are not direct activators of sirt1. *J. Biol. Chem*. 285 8340–8351. 10.1074/jbc.m109.088682 20061378 PMC2832984

[B90] Paneni F. Volpe M. Luscher T. F. Cosentino F. (2013). Sirt1, p66(shc), and set7/9 in vascular hyperglycemic memory: bringing all the strands together. *Diabetes* 62 1800–1807. 10.2337/db12-1648 23704521 PMC3661615

[B91] Park S. J. Ahmad F. Philp A. Baar K. Williams T. Luo H. (2012). Resveratrol ameliorates aging-related metabolic phenotypes by inhibiting camp phosphodiesterases. *Cell* 148 421–433. 10.1016/j.cell.2012.01.017 22304913 PMC3431801

[B92] Piechota-Polanczyk A. Kopacz A. Kloska D. Zagrapan B. Neumayer C. Grochot-Przeczek A. (2018). Simvastatin treatment upregulates ho-1 in patients with abdominal aortic aneurysm but independently of nrf2. *Oxidat. Med. Cell. Longevity* 2018:2028936. 10.1155/2018/2028936 PMC5883937 29743974

[B93] Posa A. Kupai K. Menesi R. Szalai Z. Szabo R. Pinter Z. (2013). Sexual dimorphism of cardiovascular ischemia susceptibility is mediated by heme oxygenase. *Oxidat. Med. Cell. Longevity* 2013:521563. 10.1155/2013/521563 PMC3791627 24163720

[B94] Potente M. Ghaeni L. Baldessari D. Mostoslavsky R. Rossig L. Dequiedt F. (2007). Sirt1 controls endothelial angiogenic functions during vascular growth. *Genes Dev*. 21 2644–2658. 10.1101/gad.435107 17938244 PMC2000327

[B95] Quintana R. A. Taylor W. R. (2019). Cellular mechanisms of aortic aneurysm formation. *Circ. Res*. 124 607–618. 10.1161/circresaha.118.313187 30763207 PMC6383789

[B96] Radak Z. Koltai E. Taylor A. W. Higuchi M. Kumagai S. Ohno H. (2013). Redox-regulating sirtuins in aging, caloric restriction, and exercise. *Free Radic. Biol. Med*. 58 87–97. 10.1016/j.freeradbiomed.2013.01.004 23339850

[B97] Rateri D. L. Moorleghen J. J. Balakrishnan A. Owens A. P. III Howatt D. A. Subramanian V. (2011). Endothelial cell-specific deficiency of ang ii type 1a receptors attenuates ang ii-induced ascending aortic aneurysms in ldl receptor-/- mice. *Circ. Res*. 108 574–581. 10.1161/circresaha.110.222844 21252156 PMC3076204

[B98] Robinet P. Milewicz D. M. Cassis L. A. Leeper N. J. Lu H. S. Smith J. D. (2018). Consideration of sex differences in design and reporting of experimental arterial pathology studies-statement from atvb council. *Arterioscler. Thromb. Vasc. Biol*. 38 292–303. 10.1161/atvbaha.117.309524 29301789 PMC5785439

[B99] Rodriguez F. Lamon B. D. Gong W. Kemp R. Nasjletti A. (2004). Nitric oxide synthesis inhibition promotes renal production of carbon monoxide. *Hypertension* 43 347–351. 10.1161/01.hyp.0000111721.97169.97 14698998

[B100] Rodriguez F. Lopez B. Perez C. Fenoy F. J. Hernandez I. Stec D. E. (2011). Chronic tempol treatment attenuates the renal hemodynamic effects induced by a heme oxygenase inhibitor in streptozotocin diabetic rats. *Am. J. Physiol. Regul. Integr. Comp. Physiol*. 301 R1540–R1548. 21849637 10.1152/ajpregu.00847.2010

[B101] Rodriguez F. Nieto-Ceron S. Fenoy F. J. Lopez B. Hernandez I. Martinez R. R. (2010). Sex differences in nitrosative stress during renal ischemia. *Am. J. Physiol. Regul. Integr. Comp Physiol*. 299 R1387–R1395. 20702799 10.1152/ajpregu.00503.2009

[B102] Rodriguez F. Zhang F. Dinocca S. Nasjletti A. (2003). Nitric oxide synthesis influences the renal vascular response to heme oxygenase inhibition. *Am. J. Physiol. Renal. Physiol*. 284 F1255–F1262. 12736167 10.1152/ajprenal.00435.2002

[B103] Salom M. G. Ceron S. N. Rodriguez F. Lopez B. Hernandez I. Martinez J. G. (2007). Heme oxygenase-1 induction improves ischemic renal failure: role of nitric oxide and peroxynitrite. *Am. J. Physiol. Heart Circ. Physiol*. 293 H3542–H3549. 17890422 10.1152/ajpheart.00977.2007

[B104] Sartori-Valinotti J. C. Iliescu R. Fortepiani L. A. Yanes L. L. Reckelhoff J. F. (2007). Sex differences in oxidative stress and the impact on blood pressure control and cardiovascular disease. *Clin. Exp. Pharmacol. Physiol*. 34 938–945. 10.1111/j.1440-1681.2007.04643.x 17645644

[B105] Schillinger M. Exner M. Mlekusch W. Domanovits H. Huber K. Mannhalter C. (2002). Heme oxygenase-1 gene promoter polymorphism is associated with abdominal aortic aneurysm. *Thromb. Res*. 106 131–136. 10.1016/s0049-3848(02)00100-7 12182912

[B106] Shao D. Fry J. L. Han J. Hou X. Pimentel D. R. Matsui R. (2014). A redox-resistant sirtuin-1 mutant protects against hepatic metabolic and oxidant stress. *J. Biol. Chem*. 289 7293–7306. 10.1074/jbc.m113.520403 24451382 PMC3953247

[B107] Shao D. Han J. Hou X. Fry J. Behring J. B. Seta F. (2017). Glutaredoxin-1 deficiency causes fatty liver and dyslipidemia by inhibiting sirtuin-1. *Antioxid. Redox Signal*. 27 313–327. 10.1089/ars.2016.6716 27958883 PMC5563925

[B108] Singh K. Bonaa K. H. Jacobsen B. K. Bjork L. Solberg S. (2001). Prevalence of and risk factors for abdominal aortic aneurysms in a population-based study: the tromso study. *Am. J. Epidemiol*. 154 236–244. 10.1093/aje/154.3.236 11479188

[B109] Siu K. L. Li Q. Zhang Y. Guo J. Youn J. Y. Du J. (2017). Nox isoforms in the development of abdominal aortic aneurysm. *Redox Biol*. 11 118–125. 10.1016/j.redox.2016.11.002 27912196 PMC5133668

[B110] Sodhi K. Puri N. Favero G. Stevens S. Meadows C. Abraham N. G. (2015). Fructose mediated non-alcoholic fatty liver is attenuated by ho-1-sirt1 module in murine hepatocytes and mice fed a high fructose diet. *PLoS One* 10:e0128648. 10.1371/journal.pone.0128648 26098879 PMC4476565

[B111] Sosnowska B. Mazidi M. Penson P. Gluba-Brzozka A. Rysz J. Banach M. (2017). The sirtuin family members sirt1, sirt3 and sirt6: their role in vascular biology and atherogenesis. *Atherosclerosis* 265 275–282. 10.1016/j.atherosclerosis.2017.08.027 28870631

[B112] Strycharz J. Rygielska Z. Swiderska E. Drzewoski J. Szemraj J. Szmigiero L. (2018). Sirt1 as a therapeutic target in diabetic complications. *Curr. Med. Chem*. 25 1002–1035. 10.2174/0929867324666171107103114 29110598

[B113] Suttner D. M. Dennery P. A. (1999). Reversal of ho-1 related cytoprotection with increased expression is due to reactive iron. *FASEB J*. 13 1800–1809. 10.1096/fasebj.13.13.1800 10506583

[B114] Tai H. C. Tsai P. J. Chen J. Y. Lai C. H. Wang K. C. Teng S. H. (2016). Peroxisome proliferator-activated receptor gamma level contributes to structural integrity and component production of elastic fibers in the aorta. *Hypertension* 67 1298–1308. 10.1161/hypertensionaha.116.07367 27045031 PMC4865434

[B115] Thompson A. M. Wagner R. Rzucidlo E. M. (2014). Age-related loss of sirt1 expression results in dysregulated human vascular smooth muscle cell function. *Am. J. Physiol. Heart Circ. Physiol*. 307 H533–H541. 24973384 10.1152/ajpheart.00871.2013

[B116] Thorup C. Jones C. L. Gross S. S. Moore L. C. Goligorsky M. S. (1999). Carbon monoxide induces vasodilation and nitric oxide release but suppresses endothelial nos. *Am. J. Physiol*. 277 F882–F889. 10600935 10.1152/ajprenal.1999.277.6.F882

[B117] van Andel M. M. Groenink M. Zwinderman A. H. Mulder B. J. M. de Waard V. (2019). The potential beneficial effects of resveratrol on cardiovascular complications in marfan syndrome patients(-)insights from rodent-based animal studies. *Int. J. Mol. Sci.* 20:1122. 10.3390/ijms20051122 30841577 PMC6429290

[B118] van der Pluijm I. Burger J. van Heijningen P. M. Aaa I. J. van Vliet N. Milanese C. (2018). Decreased mitochondrial respiration in aneurysmal aortas of fibulin-4 mutant mice is linked to pgc1a regulation. *Cardiovasc. Res*. 114 1776–1793. 10.1093/cvr/cvy150 29931197 PMC6198735

[B119] Venkatasubramanian S. Noh R. M. Daga S. Langrish J. P. Joshi N. V. Mills N. L. (2013). Cardiovascular effects of a novel sirt1 activator, srt2104, in otherwise healthy cigarette smokers. *J. Am. Heart Assoc*. 2:e000042. 10.1161/JAHA.113.000042 PMC3698759 23770971

[B120] Venkatasubramanian S. Noh R. M. Daga S. Langrish J. P. Mills N. L. Waterhouse B. R. (2016). Effects of the small molecule sirt1 activator, srt2104 on arterial stiffness in otherwise healthy cigarette smokers and subjects with type 2 diabetes mellitus. *Open Heart* 3:e000402. 10.1136/openhrt-2016-000402 27239324 PMC4879341

[B121] Virani S. S. Alonso A. Benjamin E. J. Bittencourt M. S. Callaway C. W. Carson A. P. (2020). American Heart Association Council on E. Prevention Statistics C, Stroke Statistics S. Heart disease and stroke statistics-2020 update: a report from the american heart association. *Circulation* 141 e139–e596. 31992061 10.1161/CIR.0000000000000757

[B122] Watson A. Nong Z. Yin H. O’Neil C. Fox S. Balint B. (2017). Nicotinamide phosphoribosyltransferase in smooth muscle cells maintains genome integrity, resists aortic medial degeneration, and is suppressed in human thoracic aortic aneurysm disease. *Circ. Res*. 120 1889–1902. 10.1161/circresaha.116.310022 28356339

[B123] Wicinski M. Socha M. Walczak M. Wodkiewicz E. Malinowski B. Rewerski S. (2018). Beneficial effects of resveratrol administration-focus on potential biochemical in cardiovascular conditions. *Nutrients* 10:1813. 10.3390/nu10111813 30469326 PMC6266814

[B124] Yang H. H. van Breemen C. Chung A. W. (2010). Vasomotor dysfunction in the thoracic aorta of marfan syndrome is associated with accumulation of oxidative stress. *Vasc. Pharmacol*. 52 37–45. 10.1016/j.vph.2009.10.005 19879959

[B125] Yu L. M. Dong X. Xue X. D. Zhang J. Li Z. Wu H. J. (2019). Protection of the myocardium against ischemia/reperfusion injury by punicalagin through an sirt1-nrf-2-ho-1-dependent mechanism. *Chem. Biol. Interact*. 306 152–162. 10.1016/j.cbi.2019.05.003 31063767

[B126] Zampino M. Yuzhakova M. Hansen J. McKinney R. D. Goldspink P. H. Geenen D. L. (2006). Sex-related dimorphic response of hif-1 alpha expression in myocardial ischemia. *Am. J. Physiol. Heart Circ. Physiol*. 291 H957–H964. 16603692 10.1152/ajpheart.00580.2005

[B127] Zee R. S. Yoo C. B. Pimentel D. R. Perlman D. H. Burgoyne J. R. Hou X. (2010). Redox regulation of sirtuin-1 by s-glutathiolation. *Antioxid. Redox Signal*. 13 1023–1032. 10.1089/ars.2010.3251 20392170 PMC2959181

[B128] Zeng H. T. Fu Y. C. Yu W. Lin J. M. Zhou L. Liu L. (2013). Sirt1 prevents atherosclerosis via liverxreceptor and nfkappab signaling in a u937 cell model. *Mol. Med. Rep*. 8 23–28. 10.3892/mmr.2013.1460 23652462

[B129] Zhang F. Kaide J. I. Rodriguez-Mulero F. Abraham N. G. Nasjletti A. (2001). Vasoregulatory function of the heme-heme oxygenase-carbon monoxide system. *Am. J. Hypertens*. 14 62S–67S. 11411767 10.1016/s0895-7061(01)02071-4

[B130] Zhang W. Huang Q. Zeng Z. Wu J. Zhang Y. Chen Z. (2017). Sirt1 inhibits oxidative stress in vascular endothelial cells. *Oxidative Med. Cell. Longevity* 2017:7543973. 10.1155/2017/7543973 PMC5435972 28546854

[B131] Zhang X. Thatcher S. Wu C. Daugherty A. Cassis L. A. (2015). Castration of male mice prevents the progression of established angiotensin ii-induced abdominal aortic aneurysms. *J. Vasc. Surg*. 61 767–776. 10.1016/j.jvs.2013.11.004 24439319 PMC4099302

[B132] Zhang Z. Xu J. Liu Y. Wang T. Pei J. Cheng L. (2018). Mouse macrophage specific knockout of sirt1 influences macrophage polarization and promotes angiotensin ii-induced abdominal aortic aneurysm formation. *J. Genet. Genom.* 45 25–32. 10.1016/j.jgg.2018.01.002 29396144

[B133] Zhou S. Chen H. Z. Wan Y. Z. Zhang Q. J. Wei Y. S. Huang S. (2011). Repression of p66shc expression by sirt1 contributes to the prevention of hyperglycemia-induced endothelial dysfunction. *Circ. Res*. 109 639–648. 10.1161/circresaha.111.243592 21778425

[B134] Zordoky B. N. Robertson I. M. Dyck J. R. (2015). Preclinical and clinical evidence for the role of resveratrol in the treatment of cardiovascular diseases. *Biochim. Biophys. Acta* 1852 1155–1177. 10.1016/j.bbadis.2014.10.016 25451966

[B135] Zuniga-Munoz A. M. Perez-Torres I. Guarner-Lans V. Nunez-Garrido E. Velazquez Espejel R. Huesca-Gomez C. (2017). Glutathione system participation in thoracic aneurysms from patients with marfan syndrome. *VASA Zeitschrift Gefasskrankheiten* 46 177–186. 10.1024/0301-1526/a000609 28173744

